# A Bibliometric Analysis of Study of Associations of Certain Genotypes with the Cardiovascular Form of Diabetic Neuropathy

**DOI:** 10.1155/2024/6761451

**Published:** 2024-04-15

**Authors:** Nazira B. Bekenova, Tamara A. Vochshenkova, Nurgul Ablakimova, Aliya Zhylkybekova, Nadiar M. Mussin, Rustam K. Albayev, Asset A. Kaliyev, Amin Tamadon

**Affiliations:** ^1^Gerontology Center, Medical Center of the President's Affairs Administration of the Republic of Kazakhstan, Astana, Kazakhstan; ^2^Department of Pharmacology, West Kazakhstan Marat Ospanov Medical University, Aktobe, Kazakhstan; ^3^Department of Evidence-Based Medicine and Scientific Management, West Kazakhstan Marat Ospanov Medical University, Aktobe, Kazakhstan; ^4^General Surgery, West Kazakhstan Marat Ospanov Medical University, Aktobe, Kazakhstan; ^5^Department for Natural Sciences, West Kazakhstan Marat Ospanov Medical University, Aktobe, Kazakhstan; ^6^PerciaVista R&D Co., Shiraz, Iran

## Abstract

This bibliometric analysis explores the landscape of research on the associations between specific genotypes and the cardiovascular form of diabetic neuropathy. Diabetes mellitus (DM) is a major contributor to premature mortality, primarily due to increased susceptibility to cardiovascular diseases. The global prevalence of DM is rising, with projections indicating further increases. Diabetic neuropathy, a complication of DM, includes the cardiovascular subtype, posing challenges in diagnosis and management. Understanding the genetic basis of cardiovascular diabetic neuropathy is crucial for targeted therapeutic interventions. The study utilizes bibliometric analysis to synthesize existing literature, identify trends, and guide future research. The Scopus database was searched, applying inclusion criteria for English articles related to genotypes and cardiovascular diabetic neuropathy. The analysis reveals a dynamic field with a notable impact, collaborative efforts, and multidimensional aspects. Publication trends over 1997-2023 demonstrate fluctuating research intensity. Top journals, authors, and affiliations are highlighted, emphasizing global contributions. Keyword analysis reveals thematic trends, and citation analysis identifies influential documents. Limitations include database biases, incomplete metadata, and search query specificity. The urgent need to explore genetic factors in cardiovascular diabetic neuropathy aligns with the increasing global diabetes burden. This analysis provides a comprehensive overview, contributing to the broader discourse on diabetic neuropathy research.

## 1. Introduction

Diabetes mellitus (DM) has emerged as a leading contributor to premature mortality in many nations, primarily due to an elevated susceptibility to cardiovascular diseases, accounting for 50 to 80% of fatalities among diabetic individuals [1]. The escalating prevalence of diabetes is attributed to factors such as population expansion, aging, the continuous rise in obesity, and sedentary lifestyles. In 2017, the global incidence, prevalence, disability-adjusted deaths, and years of quality life lost due to DM were recorded at 22.9 million, 476.0 million, 1.37 million, and 67.9 million, respectively. Projections for 2025 anticipate a further rise to 26.6 million incidences, 570.9 million cases, 1.59 million disability-adjusted deaths, and 79.3 million years of quality life lost [2].

The progression of diabetes is intricately linked to chronic complications encompassing cardiomyopathy, retinopathy, nephropathy, and neuropathy [3]. Diabetic neuropathy, characterized by damaged nervous fibers, notably triggers dysfunction in the autonomic nervous system, giving rise to cardiovascular autonomic neuropathy (CAN) [4].

Diabetic neuropathy, a debilitating complication of diabetes mellitus, manifests in various forms, including the cardiovascular subtype, which poses significant challenges in both diagnosis and management [5]. Understanding the genetic underpinnings of diabetic neuropathy, particularly its cardiovascular manifestations, is crucial for advancing targeted therapeutic interventions and improving patient outcomes.

The development of CAN in individuals with diabetes is influenced by a complex interplay of genetic and environmental factors. While specific genes associated with the development of CAN may vary among individuals, several candidate genes have been studied in the context of diabetic neuropathy and its cardiovascular manifestations. It is important to note that research in this area is ongoing, and the identification of specific genes and their roles in cardiovascular neuropathy is still a subject of investigation. Some of the potential gene candidates include polymorphisms in diabetes-related genes, inflammatory and immune response genes, neurotrophic factor genes, endothelial function-related genes, genes involved in oxidative stress pathways, and genes associated with nerve regeneration [6, 7].

While individual studies have explored the associations between specific genotypes and diabetic neuropathy, there is a need for a comprehensive synthesis of the existing literature to discern overarching trends, identify knowledge gaps, and guide future research directions. This bibliometric analysis is aimed at filling this void by systematically examining the landscape of publications on the associations of certain genotypes with the cardiovascular form of diabetic neuropathy. Bibliometric analysis, a quantitative method for evaluating and summarizing research output within a specific field, allows us to gain insights into the evolving trends, key contributors, and thematic clusters within the literature [8]. By undertaking such an analysis, we seek to provide a holistic overview of the current state of knowledge, fostering a deeper understanding of the genetic aspects of cardiovascular diabetic neuropathy.

The urgency of this endeavor is underscored by the increasing prevalence of diabetes globally and the associated rise in diabetic complications [9]. A thorough exploration of the genetic factors contributing to the cardiovascular manifestation of diabetic neuropathy can pave the way for personalized medicine approaches and targeted interventions [10]. In this context, this article outlines the objectives, methods, and anticipated contributions of the proposed bibliometric analysis, aiming to contribute to the broader discourse on diabetic neuropathy research and its translational implications.

## 2. Materials and Methods

### 2.1. Data Source

The Scopus database was employed as the primary data source for this bibliometric analysis. The search was conducted in January 2024 to retrieve relevant articles. Scopus, a comprehensive multidisciplinary abstract and citation database, was chosen for its extensive coverage of scientific literature [11].

### 2.2. Inclusion and Exclusion Criteria

To ensure the relevance and quality of the retrieved articles, the following inclusion criteria were applied: English language articles and articles related to the associations of specific genotypes with cardiovascular diabetic neuropathy. The following exclusion criteria were applied:

- *Articles not meeting inclusion criteria*: articles that did not focus on the associations of specific genotypes with cardiovascular diabetic neuropathy were excluded. This criterion ensured that only relevant articles were included in the analysis
- *Articles not available in full text*: only articles that were available in full text were included in the analysis. Abstracts or summaries without full-text access were excluded to ensure that the analysis was based on complete information
- *Duplicate articles*: duplicate articles from the same or different databases were excluded to avoid duplication of data and ensure that each unique article was counted only once in the analysis
- *Conference abstracts without full peer review*: articles that were published as conference abstracts and did not undergo full peer review were excluded. This criterion ensured that only articles that had undergone rigorous peer review were included in the analysis
- *Non-English language articles*: articles that were not written in English were excluded from the analysis. This criterion was applied to ensure consistency in language and facilitate data extraction and analysis

### 2.3. Query Formulation

**Figure 1:**
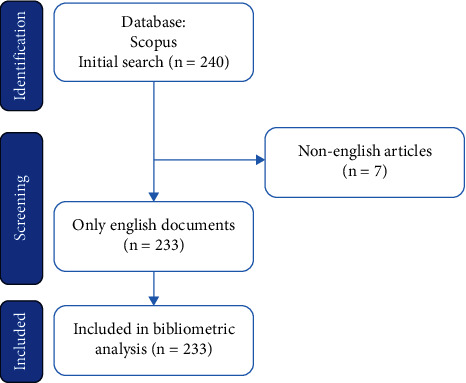
The flow chart of the screening process using PRISMA.

A search strategy was developed to identify articles meeting the inclusion criteria. The search query comprised a combination of relevant keywords and Boolean operators. For the bibliometric analysis, a carefully crafted search query is imperative, involving the selection of pertinent keywords and the construction of a search string that encapsulates the essence of the research topic. The specific query used for this study is detailed in Table 1, aimed at retrieving articles that explore the associations of certain genotypes with the cardiovascular form of diabetic neuropathy, with a focus on relevant terms such as diabetic neuropathy, cardiovascular autonomic neuropathy, genotypes, genetic polymorphism, and gene association. To visually depict the data extraction procedure, a PRISMA flowchart illustrating the selection process is provided in Figure 1.

### 2.4. R Studio and Biblioshiny

The bibliometric analysis was performed using R Studio version 2023.06.1, a powerful open-source statistical software, and the Biblioshiny tool version 4.1.4. Biblioshiny facilitates interactive and user-friendly bibliometric analyses, enabling the exploration of publication trends, collaboration networks, and keyword associations [12]. Coauthorship networks were constructed to identify collaborative patterns among researchers. Keyword cooccurrence analysis was conducted to identify clusters of terms indicative of research themes. Citation patterns were analyzed to determine the impact and influence of articles within the field.

## 3. Results

### 3.1. Overview of Retrieved Articles

The bibliometric analysis reveals a dynamic and growing field, with an impressive annual growth rate. The documents, spanning a substantial timeframe, reflect an average document age of 9.14 years, indicating ongoing interest and relevance. The high average citations per document (82.54) suggest that the research in this area has a notable impact. Collaboration is a prevalent aspect, with an average of 7.39 coauthors per document, and nearly 20% of collaborations are international. The diversity of keywords and substantial author involvement underscores the multidimensional nature of the field. Overall, the dataset provides a rich resource for understanding the trends, collaborations, and impact within the scope of the analyzed bibliometric data. These findings highlight the vibrant nature of research in this field and underscore the importance of continued collaboration and exploration in understanding the complexities of associations between specific genotypes and the cardiovascular form of diabetic neuropathy.

### 3.2. Publication Distribution over Time

**Figure 2:**
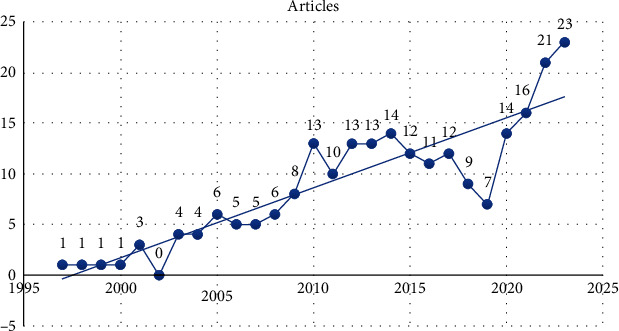
Publication distribution over 1997-2023 on associations of different genotypes with the cardiovascular form of diabetic neuropathy.

The analysis of publication trends over the timespan from 1997 to 2023 reveals a dynamic evolution in the exploration of associations between specific genotypes and the cardiovascular form of diabetic neuropathy.  Figure 2 illustrates the fluctuating intensity of research activity throughout these years. Notable peaks and troughs may signify periods of heightened interest, while a steady or increasing trend may indicate sustained attention to the subject matter. The distribution over time provides a valuable insight into the chronological development of research in this domain, allowing for the identification of key periods of growth and potential shifts in focus. Researchers and stakeholders can utilize this information to contextualize findings, identify emerging themes, and comprehend the trajectory of scholarly interest in the genetic aspects of cardiovascular diabetic neuropathy. These trends highlight the dynamic nature of research in this field, suggesting evolving interests and priorities that shape the direction of scholarly inquiry.

### 3.3. Journals and Publication Outlets

Table 2 presents a comprehensive overview of the top journals contributing significantly to the discourse on the associations between specific genotypes and the cardiovascular form of diabetic neuropathy. International Journal of Molecular Sciences emerges as a prominent source with seven articles, highlighting its pivotal role in disseminating research in this domain. Following closely are Frontiers in Endocrinology and Diabetes Care, each with six and five articles, respectively. These journals, alongside other impactful outlets such as Diabetologia and Current Pharmaceutical Design, play a crucial role in shaping the scholarly landscape. These findings underscore the importance of these journals in advancing knowledge in the field and suggest key avenues for researchers to disseminate their work and engage with the scholarly community.

### 3.4. Authorship and Collaboration

**Figure 3:**
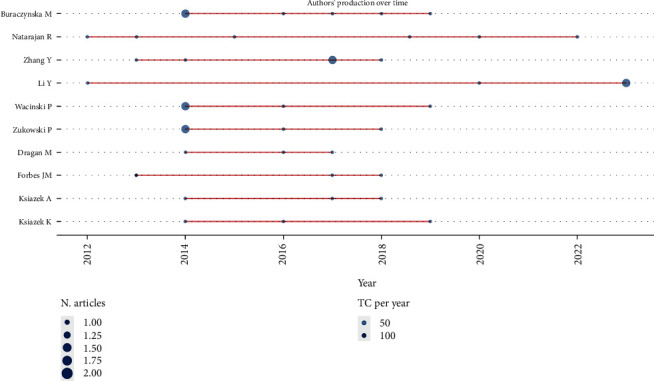
Top 10 authors and their production over time (1997-2003).

Buraczynska M emerges as the most prolific author, having authored six articles, demonstrating a significant and sustained commitment to the subject. Following closely are Natarajan R and Zhang Y, each with five articles, underlining their noteworthy impact in this research domain. Other notable contributors include Li Y, Wacinski P, and Zukowski P, each with four articles. This comprehensive list reflects the diversity of researchers actively engaging with the genetic aspects of cardiovascular diabetic neuropathy.  Figure 3 provides a visual representation of the publication output of these prolific authors, offering insights into their collective impact and individual contributions to the evolving landscape of this field. These prolific authors, along with others in the field, contribute significantly to the breadth and depth of research on the genetic aspects of cardiovascular diabetic neuropathy, highlighting the collaborative nature of scientific inquiry in this area.

Harvard Medical School and Medical University of Lublin emerge as the leading contributors, each affiliated with 13 articles, indicating their substantial impact on the field. Affiliated Hospital of Jiangnan University and University of Leipzig closely follow with seven articles each, showcasing their noteworthy contributions. Other influential affiliations include Tehran University of Medical Sciences, Texas Tech University Health Sciences Center, University of Pittsburgh, “Victor Babeș” University of Medicine and Pharmacy, Ege University, and The First Affiliated Hospital of Nanchang University, each affiliated with four to six articles (Table 3). These affiliations represent a global network of researchers and institutions contributing significantly to the understanding of the genetic aspects of cardiovascular diabetic neuropathy, highlighting the diverse and international nature of research in this field.

**Figure 4:**
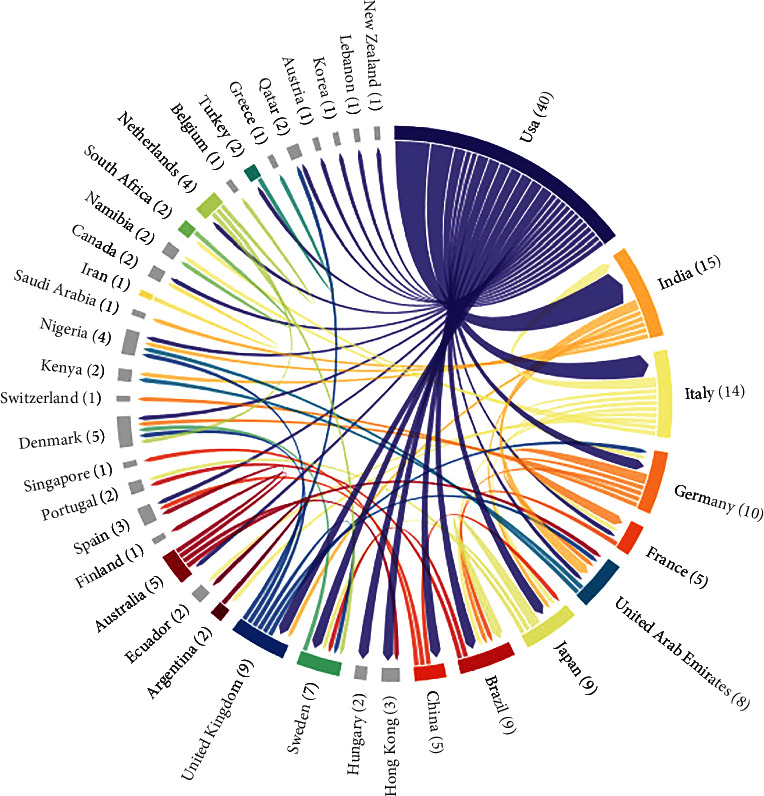
Country collaboration (1997-2023) on the topic of associations of certain genotypes with the cardiovascular form of diabetic neuropathy.

The United States emerges as a significant global contributor, producing 147 articles, followed by China, Italy, and India with 83, 43, and 42 articles, respectively. Other notable contributors include the United Kingdom, Japan, Turkey, Australia, Germany, and South Korea, each making substantial contributions ranging from 21 to 37 articles. The diverse representation of countries signifies a global collaboration in advancing knowledge on the genetic aspects of cardiovascular diabetic neuropathy.  Figure 4 illustrates the distribution of research output across different regions in the study of associations between specific genotypes and the cardiovascular form of diabetic neuropathy. This figure visually captures the collaborative networks between countries, providing a comprehensive overview of international collaboration patterns and highlighting the interconnected nature of research efforts in this critical field. These findings underscore the global significance of research on the genetic aspects of cardiovascular diabetic neuropathy, with contributions from a diverse range of countries. The collaborative nature of this research, as illustrated in Figure 4, emphasizes the importance of international cooperation in advancing our understanding of this complex condition.

### 3.5. Keyword Analysis

**Figure 5:**
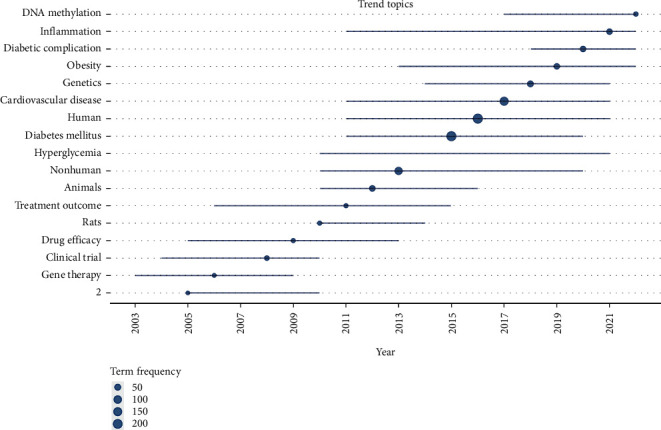
The timeline of the trend topics. Each bubble corresponds to the peak frequency of usage for a specific theme, and the connecting line signifies the years during which the theme was prominently employed.

The keyword analysis uncovered several prominent trends indicative of the evolving landscape in the study of associations between specific genotypes and the cardiovascular form of diabetic neuropathy. Notable keywords and their frequency distributions shed light on key thematic areas, offering valuable insights into the prevailing research interests over time. Gene therapy emerges as a recurring focus, with 14 instances identified. The distribution over time indicates increased attention from 2003 to 2009, suggesting a sustained interest in exploring genetic interventions. The keyword “drug efficacy” is a prevalent theme, appearing 18 times. The upward trajectory from 2005 to 2013 signifies a sustained commitment to investigating the effectiveness of pharmacological interventions. Research involving rats shows consistency, with 19 occurrences. The stable presence from 2010 to 2014 highlights the ongoing use of rodent models to study various aspects of diabetic neuropathy. “Treatment outcome” emerges as a key consideration, with 16 instances. The timeline indicates a consistent focus from 2006 to 2015, emphasizing the importance of evaluating the overall impact of interventions. Hyperglycemia and diabetes mellitus, pivotal elements of diabetic neuropathy, feature prominently with 68 and 237 occurrences, respectively. The timeline reflects sustained attention, underlining their central role in research. Cardiovascular disease is a focal point, with 168 occurrences. The timeline demonstrates a steady increase from 2011 to 2021, highlighting the recognition of cardiovascular implications in diabetic neuropathy. The keyword “genetics” appears 65 times, with a continuous rise from 2014 to 2021, showcasing an increasing focus on genetic aspects within the broader context of diabetic neuropathy. The theme of “obesity” is evident with 56 occurrences. The timeline indicates sustained interest, particularly from 2013 to 2022, emphasizing the intersection of obesity and diabetic neuropathy. “Diabetic complication” is a notable keyword with 36 occurrences, displaying a significant presence from 2018 to 2022, underscoring the growing attention to complications arising from diabetic neuropathy. DNA methylation, a specific molecular aspect, appears 20 times, with a notable rise from 2017 to 2022, signifying an increasing interest in epigenetic mechanisms within the field (Figure 5).

The keyword analysis revealed prominent trends in the study of associations between specific genotypes and the cardiovascular form of diabetic neuropathy. Notable keywords and their frequency distributions provided insights into prevailing research interests over time. For example, “gene therapy” emerged as a recurring focus, suggesting sustained interest in exploring genetic interventions. Similarly, the keyword “drug efficacy” showed a consistent upward trajectory, indicating a commitment to investigating pharmacological interventions. These findings highlight the evolving landscape of research in this area, reflecting a multifaceted approach to understanding and addressing diabetic neuropathy.

### 3.6. Citation Analysis

The high average citations per document (82.54) suggest that the research in this area has a notable impact. In our analysis, three highly influential documents were identified, each amassing over one hundred citations, signifying their substantial impact within the scholarly community. Giacco's work, published in Circulation Research in 2010, has garnered an impressive 3786 citations, attesting to its enduring significance in the field. Zheng's contribution in Nature Reviews Endocrinology from 2018 is another noteworthy document, with 2892 citations, underscoring its widespread recognition and influence. Furthermore, Forbes's publication in Physiology Reviews in 2013 has accumulated 1756 citations, emphasizing its enduring impact and contribution to the academic discourse. These highly cited documents serve as pillars in the literature, shaping and guiding the discourse on associations between specific genotypes and the cardiovascular form of diabetic neuropathy (Table 4).

The citation analysis highlights the significant impact of key studies in the field. For instance, Giacco's research in Circulation Research from 2010, with 3786 citations, underscores its lasting relevance. Similarly, Zheng's work in Nature Reviews Endocrinology from 2018 has 2892 citations, indicating its wide recognition. Forbes's publication in Physiology Reviews in 2013, with 1756 citations, also demonstrates its enduring influence. These studies are pivotal in shaping our understanding of the genetic aspects of cardiovascular diabetic neuropathy.

### 3.7. Limitations of the Analysis

It is essential to acknowledge potential limitations in the obtained results to ensure a comprehensive understanding of the study's scope and implications. One notable limitation lies in the reliance on bibliometric data, which may introduce biases based on the coverage and indexing practices of the selected database, in this case, Scopus. Variations in database coverage could influence the representation of articles and potentially overlook relevant publications in nonindexed sources. Additionally, the bibliometric analysis relies heavily on the accuracy of metadata, and discrepancies or incomplete information in the database may introduce data gaps. Methodological constraints include the specificity of the search query, which might inadvertently exclude relevant articles with alternative terminologies or keywords. Furthermore, the analysis primarily focuses on quantitative metrics and may not capture nuanced qualitative aspects of the research.

## 4. Discussion

The dynamic evolution of publication trends highlights periods of heightened interest, possibly reflecting advancements in technology and research methodologies. Collaboration is evident, emphasizing the interdisciplinary nature of the field. Noteworthy journals, prolific authors, and impactful affiliations underscore the diverse contributors shaping the scholarly landscape. The geographic distribution reveals a global collaboration, fostering a comprehensive understanding of genetic aspects.

The keyword analysis identifies thematic areas, such as gene therapy, drug efficacy, and cardiovascular disease, illustrating the multifaceted exploration of diabetic neuropathy. The citation analysis highlights influential documents, shaping the discourse and guiding future research. The discussion delves into the significance of highly cited works and their enduring impact on the field.

Such kind of analysis plays a pivotal role in elucidating the significance of research endeavors within a specific domain. By systematically evaluating and summarizing research output, it offers valuable insights into evolving trends, influential contributors, and thematic clusters, thereby contributing to a deeper understanding of the knowledge landscape. In the context of our study on associations between genotypes and the cardiovascular form of diabetic neuropathy, the bibliometric analysis serves as an indispensable tool for gauging the breadth and impact of scholarly contributions. It not only provides a holistic overview of the current state of knowledge but also facilitates the identification of knowledge gaps and guides future research directions. This methodological approach underscores the importance of bibliometric analysis as a catalyst for advancing scientific inquiry and fostering informed decision-making in research and clinical practice [23, 24].

Diabetes, identified by hyperglycemia, results from diverse biological processes and genetic pathways. However, complications in diabetic patients may not solely stem from hyperglycemia. The progressive nature of complications, indirect diagnostic measures, and the impact of confounders make accurate phenotyping challenging. When initial studies highlighted variations in the susceptibility to diabetic complications among patients with seemingly similar diabetes glucose control, clinical features, and management, family studies revealed distinct differences in the incidence of both microvascular and macrovascular complications. Individuals with family members having both diabetes and complications showed higher complication rates compared to those with diabetes but without complications [25–27]. While family studies underscored genetic components in diabetes and its complications, early genetic research faced limitations. Linkage analysis failed to pinpoint loci with significant large effects, candidate gene studies were prone to false positives due to lenient statistical thresholds, and early genome-wide association studies lacked adequate sample sizes to detect the modest effect sizes inherent in complex traits. The complexity was compounded by investigating a disease within a disease—diabetes-induced micro- and macrovascular complications—characterized by intertwined risk factors, indirect disease diagnostics, and unclear disease progression.

According to Cole and Florez, challenges are amplified for diseases like end-stage kidney disease or coronary artery disease, where the risk of death can hinder event collection [16]. Most genetic studies favor healthier individuals, affecting results. Studying complications together hinders distinguishing shared and unique mechanisms. Key research needs involve larger datasets, improved phenotyping, objective diagnostic measures, quantitative microvascular system measurements, novel phenotypic groupings, and better use of longitudinal data. Complementary approaches using previously defined genetic clusters from general population studies can enhance understanding. Utilizing diverse populations in genetic studies will aid in discovering population-specific signals, fine-mapping known loci, and improving risk prediction for precision medicine. Sequencing datasets will explore the full spectrum of genetic variation, crucial for advancing drug development. Addressing these gaps is crucial amid the growing diabetes epidemic and the surge in genetic discoveries for diabetes complications.

## 5. Conclusion

The bibliometric analysis provides a comprehensive overview of the research landscape on the connections between genotypes and the cardiovascular aspect of diabetic neuropathy. The dynamic nature of the field, global collaboration, and multidimensional aspects underscore the complexity of understanding genetic contributions. Highly cited documents serve as pillars, guiding further investigations.

Despite limitations, this analysis contributes valuable insights, guiding future research directions. The urgency of addressing the global diabetes burden underscores the importance of unraveling genetic factors in cardiovascular diabetic neuropathy. The study emphasizes the need for targeted interventions and personalized medicine approaches. As the field evolves, ongoing collaborative efforts can enhance our understanding of this critical aspect of diabetic complications.

## Figures and Tables

**Table 1:** Search strategy for the Scopus.

Number	Query	Search result
#1	“Biology, Molecular” OR “Biochemical Genetic” OR “Biochemical Genetics” OR “Genes” OR “Genetic Code” OR “Genetic variations” OR “Genetic, Biochemical” OR “Genetic, Molecular” OR “Genetics, Biochemical” OR “Genetics, Molecular” OR “Genogroups” OR “Genotype” OR “Genome” OR “Genomics” OR “Genotypes” OR “Genogroup” OR “Molecular Genetic” OR “Molecular Genetics” OR “Genetic Predisposition to Disease”	5,165,023
#2	“Diabetic Neuropathies” OR “Diabetic Neuropathy” OR “Neuropathies, Diabetic” OR “Neuropathy, Diabetic” OR “Diabetic Autonomic Neuropathy” OR “Autonomic Neuropathies, Diabetic” OR “Autonomic Neuropathy, Diabetic” OR “Diabetic Autonomic Neuropathies” OR “Neuropathies, Diabetic Autonomic” OR “Neuropathy, Diabetic Autonomic” OR “Diabetic Neuralgia” OR “Diabetic Neuralgias” OR “Neuralgias, Diabetic” OR “Diabetic Neuropathy, Painful” OR “Diabetic Neuropathies, Painful” OR “Neuropathies, Painful Diabetic” OR “Neuropathy, Painful Diabetic” OR “Painful Diabetic Neuropathies” OR “Painful Diabetic Neuropathy” OR “Neuralgia, Diabetic” OR “Symmetric Diabetic Proximal Motor Neuropathy” OR “Asymmetric Diabetic Proximal Motor Neuropathy” OR “Diabetic Asymmetric Polyneuropathy” OR “Asymmetric Polyneuropathies, Diabetic” OR “Asymmetric Polyneuropathy, Diabetic” OR “Diabetic Asymmetric Polyneuropathies” OR “Polyneuropathies, Diabetic Asymmetric” OR “Polyneuropathy, Diabetic Asymmetric” OR “Diabetic Mononeuropathy” OR “Diabetic Mononeuropathies” OR “Mononeuropathies, Diabetic” OR “Mononeuropathy, Diabetic” OR “Diabetic Mononeuropathy Simplex” OR “Diabetic Mononeuropathy Simplices” OR “Mononeuropathy Simplex, Diabetic” OR “Mononeuropathy Simplices, Diabetic” OR “Simplex, Diabetic Mononeuropathy” OR “Simplices, Diabetic Mononeuropathy” OR “Diabetic Amyotrophy” OR “Amyotrophies, Diabetic” OR “Amyotrophy, Diabetic” OR “Diabetic Amyotrophies” OR “Diabetic Polyneuropathy” OR “Diabetic Polyneuropathies” OR “Polyneuropathies, Diabetic” OR “polyneuropathy, diabetic”	34,015
#3	“Abnormalities, Cardiovascular” OR “Abnormality, Cardiovascular” OR “Cardiovascular Abnormality” OR “Cardiovascular Abnormalities” OR “Cardiovascular Disease” OR “Disease, Cardiovascular” OR “Major Adverse Cardiac Events” OR “Cardiac Events” OR “Cardiac Event” OR “Event, Cardiac” OR “Adverse Cardiac Event” OR “Adverse Cardiac Events” OR “Cardiac Event, Adverse” OR “Cardiac Events, Adverse” OR “Angiocardiography” OR “Anti-Arrhythmia Agents” OR “Ballistocardiography” OR “Cardiotonic Agents” OR “Electrocardiography” OR “Myocardium” OR “Cardiac Failure” OR “Heart Decompensation” OR “Decompensation, Heart” OR “Heart Failure, Right-Sided” OR “Heart Failure, Right Sided” OR “Right-Sided Heart Failure” OR “Right Sided Heart Failure” OR “Myocardial Failure” OR “Congestive Heart Failure” OR “Heart Failure, Congestive” OR “Heart Failure, Left-Sided” OR “Heart Failure, Left Sided” OR “Left-Sided Heart Failure” OR “Left Sided Heart Failure” OR “Diastolic Heart Failures” OR “Heart Failure, Preserved Ejection Fraction” OR “Heart Failure, Normal Ejection Fraction” OR “Diastolic Heart Failure” OR “Heart Failures, Systolic” OR “Systolic Heart Failures” OR “Systolic Heart Failure” OR “Heart Failure, Reduced Ejection Fraction”	1,198,057
#4	#1 AND #2 AND #3	240

**Table 2:** Journals at the forefront of research on associations of different genotypes with the cardiovascular form of diabetic neuropathy.

Sources	Articles
International Journal of Molecular Sciences	7
Frontiers in Endocrinology	6
Diabetes Care	5
Diabetologia	5
Current Pharmaceutical Design	4
Journal of Diabetes and its Complications	4
Current Diabetes Reports	3
Current Medicinal Chemistry	3
Diabetes	3

**Table 3:** Affiliations and their contribution to research on genotypes and cardiovascular diabetic neuropathy (1997-2023).

Affiliations	Articles
International Journal of Molecular Sciences	13
Frontiers in Endocrinology	13
Diabetes Care	7
Diabetologia	7
Current Pharmaceutical Design	6
Journal of Diabetes and its Complications	5
Current Diabetes Reports	5
Current Medicinal Chemistry	5
Diabetes	4

**Table 4:** Top 10 highly cited studies on the topic of associations of certain genotypes with the cardiovascular form of diabetic neuropathy.

Rank	Study ID (references)	Title of the document	Journal name	Total citations	DOI
1	Giacco and Brownlee [13]	Oxidative stress and diabetic complications	Circulation Research	3786	10.1161/CIRCRESAHA.110.223545
2	Zheng et al. [14]	Global aetiology and epidemiology of type 2 diabetes mellitus and its complications	Nature Reviews Endocrinology	2892	10.1038/nrendo.2017.151
3	Forbes and Cooper [15]	Mechanisms of diabetic complications	Physiological Reviews	1756	10.1152/physrev.00045.2011
4	Cole and Florez [16]	Genetics of diabetes mellitus and diabetes complications	Nature Reviews Nephrology	559	10.1038/s41581-020-0278-5
5	Moran et al. [17]	Transient receptor potential channels as therapeutic targets	Nature Reviews Drug Discovery	464	10.1038/nrd3456
6	Aggarwal et al. [18]	Tocotrienols, the vitamin E of the 21st century: its potential against cancer and other chronic diseases	Biochemical Pharmacology	461	10.1016/j.bcp.2010.07.043
7	Ferroni et al. [19]	Platelet activation in type 2 diabetes mellitus	Journal of Thrombosis and Haemostasis	376	10.1111/j.1538-7836.2004.00836.x
8	Reddy et al. [20]	Epigenetic mechanisms in diabetic complications and metabolic memory	Diabetologia	346	10.1007/s00125-014-3462-y
9	Forbes and Thorburn [21]	Mitochondrial dysfunction in diabetic kidney disease	Nature Reviews Nephrology	314	10.1038/nrneph.2018.9
10	Tang et al. [22]	Aldose reductase, oxidative stress, and diabetic mellitus	Frontiers in Pharmacology	306	10.3389/fphar.2012.00087

## Data Availability

Data are contained within the article. Datasets related to this project can be obtained from corresponding authors based on a reasonable request.
